# HTLV Tax-1 and Tax-2 proteins enhance interferon regulatory factor 3 dependent promoter activation

**DOI:** 10.1186/1742-4690-11-S1-P97

**Published:** 2014-01-07

**Authors:** Erica Diani, Francesca Avesani, Giorgia Cremonese, Elisa Bergamo, Umberto Bertazzoni, Maria Grazia Romanelli

**Affiliations:** 1 Department of Life and Reproduction Sciences, Section of Biology and Genetics, University of Verona, Verona, Italy

## 

The HTLV-1 infection is known to induce an alteration of type I interferon (IFN-I) signaling since it is capable of escaping IFN-mediated immune response in vitro and Tax-1 protein modulates the expression of factors involved in the interferon signaling. In the present study we have investigated the effect of Tax-1 and Tax-2 expression on the activation of an IFN-regulatory factor 3 (IRF3) regulated promoter through the recruitment of the IFN-I upstream IKKε and TANK-binding kinase 1 (TBK1) factors, two IkB-related kinase homologues, which are essential for the activation of IRF3 pathway. We have demonstrated that both Tax-1 and Tax-2 were detectable in immuno-complexes formed by IKKε and TBK1 in HEK 293T transfected cells, but did not interact with the IRF3 factor. The presence of Tax-1 and IKKε in transfected cells resulted in a significant activation of the IRF3 regulated promoter. A similar effect was measured in the presence of Tax-1 and TBK1. We have also observed that Tax-1 mutants defective in sumoylation and ubiquitination post-translational modification were impaired in their ability to form complexes with IKKε or TBK1 and in the transactivating activity on IRF3 dependent promoter.These data provide evidence for a role of Tax proteins in the activation of IFN-I pathway, mediated by interaction with IKKε and TBK1 kinases. The effects of the Tax interaction with factors that act upstream of interferon regulatory factor IRF3 should be taken into account to further explain the IFN-mediated immune response to HTLV-1 infection. This study is funded by AIRC-Cariverona.

